# Reproductive Outcome After Laparoscopic Ovarian Endometrioma Stripping With Volumetric Hydrodissection

**DOI:** 10.1155/ogi/2586905

**Published:** 2026-02-18

**Authors:** Bogusław Gawlik, Joanna Trawińska, Andrzej Skręt, Joanna Bielatowicz, Małgorzata Gawlik, Edyta Barnaś, Joanna Skręt-Magierło

**Affiliations:** ^1^ Department of Obstetrics and Gynecology with Oncological Gynecology, Health Care Center, Dębica, 39-200, Poland; ^2^ Department of Gynecology, Oncological Gynecology and Obstetrics, University Clinical Hospital, Rzeszów, 35-055, Poland; ^3^ Faculty of Medicine, Jagiellonian University Medical College, Cracow, 31-008, Poland, cm-uj.krakow.pl; ^4^ Faculty of Health Sciences and Psychology, Collegium Medicum, University of Rzeszow, Rzeszów, 35-959, Poland, ur.edu.pl

**Keywords:** laparoscopic stripping, ovarian endometrioma, volumetric hydrodissection

## Abstract

**Introduction:**

Hydrodissection (HD) is used in surgical blunt dissection covering its two types. The first one is volumetric with application of neutral fluids to obtain exclusive separation of tissue planes. The second one is hybrid with additional effect by addition of some pharmaceutics like vasoconstrictors to neutral fluid. In most reports dealing with ovarian endometrioma stripping, the hybrid HD is used.

**Aim of the Work:**

To assess application of volumetric HD in endometrioma stripping in respect to intraoperative and postoperative data including reproductive outcome.

**Materials and Methods:**

The prospective observational study was conducted in a group of 53 women qualified for laparoscopic enucleation of endometrial cysts. The patients were operated with two methods according to surgeon choice. First method was the enucleation proceeded by volumetric HD, and in the other one, classic stripping was performed. The patients operated with these methods constituted two groups accordingly. All the patients were asked to fulfill the questionnaires dealing with their reproductive data.

**Results:**

Volumetric HD was not found to reduce surgery time, to diminish the frequency of ovarian stitching, and to reduce postoperative pain. Decrease in the AMH was lower in Group I, but it did not reach statistical significance (*p* = 0.19). Evacuation of intact endometrial cyst and removal of reduced resected tissue through endobag were more frequent in Group 1 (69.2% vs. 22.2%, *p* = 0.0006; 46.2% vs. 7.4% *p* = 0.0039, respectively). Very important fact was absence of mature ovarian cortex in all samples coming from Group 1. We did not observe the differences between the groups in reproductive outcome. Spontaneous conceiving was 63.1% in Group 1 vs. 63.6% in Group 2, and in‐time deliveries were 47.3% in Group 1 vs. 50.0% in Group 2.

**Conclusions:**

There are some positive postoperative changes after application of volumetric HD in endometrioma stripping, but it did not cause better reproduction outcome.

## 1. Introduction

Dissection is a basic maneuver in surgery. It is common element of surgical action. The term “dissection” originally primarily associated with autopsy was later applied to surgical procedures performed on living patients. Currently, sharp dissection and blunt dissection are recognized. Hydrodissection (HD) is often used as an element of blunt dissection. It involves the use of stream of water to seperate tissues and develop tissue planes.

Fluids like physiological saline are used, and it is described as volumetric HD because its exclusive effect is to create space between the tissues. On the other hand, hybrid HD uses addition of different pharmaceutical agents and it brings parallel effect—volumetric effect and vasoconstriction or anesthesia.

The above types of HD were applied in many medical branches, i.e., surgery, oncology, and diagnostic procedures. In surgical interventions, hybrid HD has been used in cholecystectomy [1], rectal mucosa harvest [2], and hernia repair [3]. In oncology, it has been used in nipple sparing mastectomy [4]. Volumetric HD was used in separation of hepatic tumors from intestines during radiofrequency treatment [5]. In gynecological surgery, volumetric HD and hybrid HD were jointly described in thirty eight reports. Nine of them are devoted to pelvic organ prolapse operation with the use of HD, prevalently hybrid [6–14]. Three works described hybrid or volumetric HD as an adjunct to stress urine incontinence surgery [15–17], while six reported the same type of HD in ectopic pregnancy [18–23]. Another application of HD mainly volumetric one was described in genital tumors [24, 25]. Pelvic pain was treated with both types of HD [26, 27], cervical cancer surgery with hybrid HD was described in one report [28], and vaginal hysterectomy was also described in one report with hybrid HD [29]. HD is widely applied is laparoscopic stripping of endometrioma. Six reports described the use of hybrid HD in ovarian endometrioma surgery; among them, three were original prospective studies [30–32], two were original retrospective studies [33, 34], and one was a case report [35]. Most of abovementioned reports analyzed the data focusing on intraoperative data, ovarian reserve, and histological assessment of tissues removed with the ovarian cyst. Only some reports included the data of the reproductive outcome. None used volumetric HD.

### 1.1. Aim of the Study

To assess application of volumetric HD in endometrioma stripping in respect to intraoperative and postoperative data including reproductive outcome.

## 2. Materials and Methods

The prospective observational study was conducted after obtaining the consent of the Bioethical Committee of University (No. 1a/01/2015). A total of 53 women qualified for laparoscopic enucleation of endometrial cysts were enrolled in the study.

Inclusion criteria were as follows: consent to participate in the study, consent to surgery, positive qualification for surgery, and presence of endometrial lesions in one or both ovaries.

Exclusion criteria were as follows: lack of consent to participate in the study, lack of consent for surgery, lack of qualifications for surgery, unconfirmed presence of endometrial lesions in the postoperative material, and need to convert laparoscopy to laparotomy.

Before the surgery, patients’ blood was collected for obligatory laboratory tests. The patients were asked to complete the part of the questionnaire intended for them investigating problems with conceiving, history of operations, and information about previous endometrioma treatment.

According to the choice of the operating surgeon, the patients were assigned to Group I, i.e., in which enucleation was performed with the use of neutral saline HD, and group II in which endometrial cyst was chosen to be removed with the use of classical stripping.

To assess intraoperative blood loss on the first postoperative day, venous blood was collected to determine the control level of hemoglobin. In addition, blood was collected for biochemical tests, determination of hormone levels, and assessing ovarian reserve. During the analysis of the postoperative specimen in pathology departments, the presence of ovarian tissue was assessed according to the Muzii et al. classification [36].

The questionnaires intended for this study were supplemented at the end of hospitalization with missing data on the surgical procedure, postoperative management, postoperative laboratory tests, and registration of complications. Within 30 days after the surgery, AMH level was assessed after the surgery to control the ovarian reserve.

Seven years after completion of the surgical procedures, the reproductive data were obtained by phone from 19 patients (73%) operated with volumetric HD and 22 patients (81.4%) operated with classic HD. The reproductive data included procreation plans after the procedure, number of pregnancies including spontaneous conceiving, and number of miscarriages.

**FIGURE 1:**
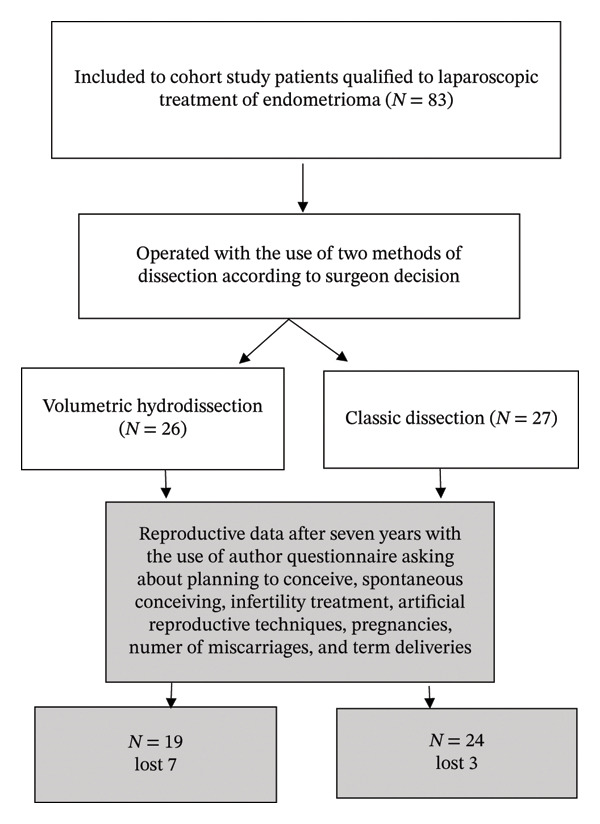
The flowchart shows the number of patients operated on using two methods: volumetric hydrodissection (*N* = 26) and classical dissection (*N* = 27). The gray rectangles present the questions about reproduction asked 7 years after surgery, as well as the number of patients from both groups who answered these questions and those who did not.

The data were developed in Statistica 8.0 software. The chi‐square test of independence was used to examine the relationship between two characteristics measured on a nominal scale (†), including Yates’s correction for continuity. Student’s *t*‐test was used for independent samples (∗). The flowchart prepared for the study (Figure 1) shows the number of patients operated on using two methods: volumetric hydrodissection (*N* = 26) and classical dissection (*N* = 27). The gray rectangles indicate the questions asked at the 7‐year follow‐up and the number of patients in both groups who responded or did not respond.

## 3. Results

Basic characteristics of the patients are presented in Table 1.

**TABLE 1:** Preoperative characteristics of studied patients (*N* = 53).

	Group I (volumetric HD) *N* = 26	Group II (classical HD) *N* = 27	*p*
Age	$\overset{¯}{x}$ = 31.0 ± 5.6	$\overset{¯}{x}$ = 32.7 ± 5.8	0.283∗
BMI	$\overset{¯}{x}$ = 22.7	$\overset{¯}{x}$ = 22.5	0.470∗
Nulliparous	14 (53.8%)	18 (66.7%)	0.340^*†*^
Cesarean section	5 (19.2%)	4 (15.3%)	0.950^*†*^
Subfertility in anamnesis	13 (50%)	17 (63%)	0.341^*†*^
AMH (ng/mL)	$\overset{¯}{x}$ = 3.34 ± 1.81	$\overset{¯}{x}$ = 3.92 ± 2.09	0.285∗

*Note:* Our study was nonrandomized, but in preoperative data, we did not find differences between the groups.

Intraoperative data are presented in Table 2.

^†^Chi‐square test of independence including Yates’s correction for continuity.

^∗^Student’s *t*‐test for independent samples.

**TABLE 2:** Intraoperative data of studied patients (*N* = 53).

	Group I (volumetric HD) *N* = 26	Group II (classical HD) *N* = 27	*p*
Surgery time (min)	$\overset{¯}{x}$ = 39.6 ± 17.7	$\overset{¯}{x}$ = 44.6 ± 17.4	0.305^∗^
Suturing	7 (26.9%)	5 (18.5%)	0.560^*†*^
Evacuation with endobag	12 (46.2%)	2 (7.4%)	0.004^*†*^
Enucleation of an intact cyst	18 (69.2%)	6 (22.2%)	0.001^*†*^

^†^Chi‐square test of independence including Yates’s correction for continuity.

^∗^Student’s *t*‐test for independent samples.

We found no differences between time of surgery and suturing. Only differences were more frequent removal of intact cyst in the volumetric HD group and more often cyst evacuation with endobag in the HD group.

Postoperative data are presented in Table 3.

**TABLE 3:** Postoperative data of studied patients (*N* = 53).

	Group I (volumetric HD) *N* = 26	Group II (classical HD) *N* = 27	*p*
AMH drop	$\overset{¯}{x}$ = 1.17 ± 0.72	$\overset{¯}{x}$ = 1.48 ± 0.97	0.192^∗^
Hemoglobin drop	$\overset{¯}{x}$ = 1.0 ± 0.5	$\overset{¯}{x}$ = 1.2 ± 1.1	0.397^∗^
Follicle absence	5/26	2/27	0.387^*†*^
Presence of all types of follicles including antral ones	0/26	3/27	0.250^*†*^
Self‐assessed pain on the first day postoperatively	$\overset{¯}{x}$ = 4.5 ± 1.5	$\overset{¯}{x}$ = 5.3 ± 1.7	0.075^∗^
Self‐assessed pain on the third day postoperatively	$\overset{¯}{x}$ = 2.4 ± 1.2	$\overset{¯}{x}$ = 2.2 ± 0.9	0.497^∗^

^†^Chi‐square test of independence including Yates’s correction for continuity.

^∗^Student’s *t*‐test for independent samples.

We found no differences in postoperative data on AMH and hemoglobin drop, as well as the self‐assessed intensity of pain during the postoperative period.

No differences were found in histological assessment of ovarian tissues removed with the cyst. However, it was worth noting that in the volumetric HD group, no specimens were found with ovarian tissue with all types of follicles including mature ovarian cortex tissue.

Reproductive outcome 7 years after the surgery is presented in Table 4 and graphically in Figure S1 in supporting information in response two groups: volumetric HD (*N* = 19) and classical HD (*N* = 22).

**TABLE 4:** Reproductive outcome seven years after surgery.

	Group I (volumetric HD) *N* = 19 (%)	Group II (classical HD) *N* = 22 (%)	*p*
Planning to conceive	15/78.9	19/86.3	0.832^∗^
Spontaneous conceiving	12/63.1	14/63.6	0.975^∗^
Infertility treatment	11/57.8	17/77.2	0.184^∗^
ART	6/31.5	13/59.0	0.078^∗^
Pregnancy	13/68.4	19/86.3	0.314^∗^
Number of miscarriages	4/21.0	8/36.3	0.465^∗^
Term deliveries	9/47.3	11/50	0.867^∗^

^∗^Student’s *t*‐test for independent samples.

We did not observe differences between the groups in terms of reproductive outcome.

## 4. Discussion

The presented study on volumetric HD in laparoscopic ovarian endometrioma stripping is unique. There are only few studies available dealing with surgery for ovarian endometrioma with HD. The vast majority of them assess the impact of the HD technique using vasopressin, i.e., hybrid HD, on improving intraoperative parameters, i.e., blood loss and/or the number of cases of cauter use [30–33, 35]. According to the cited authors, this is related to the use of vasopressin. Only two reports analyzed three groups: laparoscopic cystectomy with stripping without HD (control group), with volumetric HD with injection of saline solution, and hybrid HD connecting volumetric effect with vasopressin action [31, 32]. Both studies analyze cauterization event number, showing that the use of vasopressin significantly improves this parameter. Moreover, Qion‐Zhen et al. [31] showed that in the volumetric HD with saline use when compared with stripping without HD, fewer cauterization events were required to achieve hemostasis, and lower preoperative FSH levels were detected. In the above work, volumetric HD is analyzed as in our study. Moreover, the authors additionally analyzed the FSH level with long‐term follow‐up 3, 6, and 12 months after the procedure. In our work, we only analyzed the level of blood loss by measuring hemoglobin, but we did not observe differences between groups. Moreover, we did not assess the FSH level, only the AMH level one month after surgery. In our study, we did not observe any differences in the AMH level measured one month after surgery. A different result was obtained by Xin et al. [33], who, just one month after the surgery, found a remarkable decrease of AMH level, and the values were the lowest among different time points. AMH levels in vasopressin group were significantly higher than those in control group at each time point postoperatively [33]. In turn, Albrozi et al. [30] analyzed serum AMH concentration at 3, 6, and 12 months after surgery and did not find any significant difference between both groups (with vasopressin and control group). A different result was found by Zhang et al., who achieved normalization of AMH concentration 6 months after the procedure in a 26‐year‐old patient with a 6‐cm ovarian cyst after using “water jet” injection with vasopressin [35]. The results of the work by Cabiscuelas et al. seem interesting [34] because the authors analyzed the AMH level in two groups: endometrial cysts and nonendometrial cysts. The use of vasopressin during endometriotic cyst enucleation showed no significant impact on postoperative AMH level decline at 10 days and at 3 months follow‐up compared with the control group [34]. Current systematic review and meta‐analysis performed by Moreno‐Sepulveda et al. indicated a significant decrease in short‐ (no later than month), medium‐ (one and six months), and long‐term (six or more months) postoperative AMH levels when compared with baseline AMH. However, there were no differences between short‐ and long‐term postoperative AMH levels. A significant decrease in postoperative AMH was observed in bilateral endometriomas compared with unilateral cases. In addition, patients with endometriomas presented a significant decline in postoperative AMH compared with patients with other benign ovarian conditions. We note that these results should, however, be interpreted very carefully due to the heterogeneity of the studies included in the analyses [37]. Therefore, the available data are not consistent and do not allow to clearly determine the impact of cystectomy on postoperative parameters regarding AMH levels.

Moreover, it should be noted that despite numerous studies, it is still not clear whether ovarian endometriosis per se damages the ovarian reserve, or whether there is a relationship between ovarian endometrioma size and its effects on the ovarian reserve [38, 39]. Muzii et al. found in their meta‐analysis that patients with ovarian endometrioma not undergoing surgery have lower AMH levels compared to patients with nonendometrial cysts or healthy controls, suggesting that the mere presence of ovarian endometrioma may reduce the ovarian reserve [39]. Additionally, some evidence has been reported as to the possibility that the presence of the ovarian endometrioma per se, and not only its excision, may be detrimental to the ovarian reserve [40].

Another important parameter that was the object of our long‐term observation was reproductive outcome. Only in our study on the use of volumetric HD, selected parameters of reproductive outcome were analyzed in such a long time follow‐up. The assessment of reproductive outcome was performed in other reports regarding the removal of ovarian endometrioma at various time follow‐ups, the longest being 34 months [41]. In our study, it was a longer period, that is, 7 years. However, there is a great difficulty in comparing our results with other reports. Only in our study, we analyzed the reproductive outcome of women who underwent cystectomy with the volumetric HD. Supermaniam et al. studied the reproductive outcome in a group of 143 women treated with hybrid HD using vasopressin. Among 76 patients with preoperative infertility and with pregnancy intention, 38 (50%) were successful in conceiving. A total of 32 patients conceived spontaneously within a mean of 6.9 months. In our group of 41 patients, 26 of whom underwent volumetric HD and 27 only stripping, 68.4% vs. 86.3% conceived during 7‐year follow‐up. However, it should be noted that in the classical stripping group, ART techniques and infertility treatment were more frequently used. In our study, 21% of miscarriages were observed after volumetric HD and 36.3% in the traditional stripping group. In Supermanian et al.’s study, miscarriages occurred in only 2.6% [42]. In turn, Taniguchi et al. reported a 50% pregnancy rate (of which 50% were spontaneous) following ovarian endometrioma cystectomy [43]. In Taniguchi et al.’s study, the pregnancy rate post ovarian endometrioma cystectomy was 77.4% (24 out of 31 patients). In Kovačević’s study, the rate of spontaneous conceptions was 75% (18 out of 24) [44]. In another study, Chen et al. compared reproductive data of the group subjected to laparoscopic cystectomy (Group 1, *n* = 46) and laparoscopic ovarian drainage and ablation with bipolar coagulation at low power (Group 2, *n* = 30) and found that the pregnancy rate in Group 1 was at 71.05% and 73.08% in Group 2, with a mean follow‐up of 30.40 months and 32.35 months (*p* > 0.99), respectively [41]. Also, the analysis of two randomized trials from the Cochrane Database by Hart et al. showed a beneficial effect of excisional surgery over drainage or ablation of an endometrioma in achieving a spontaneous pregnancy in subfertile women. However, this can lead to a significant reduction in the number of ovarian follicles, especially in women who have undergone previous ovarian surgery, and therefore diminished ovarian reserve, reflected by a sustained decrease in AMH levels [45]. Hamdy et al. in the study of forty infertile women in reproductive age subjected to laparoscopic cystectomy and stripping for symptomatic ovarian endometrioma(s) observed a significant positive correlation between AMH and AFC preoperatively and after 3 months. The ovulation rate was 52.5% at 3 months, with a pregnancy rate of 42.5% [46]. It is difficult to fully compare the parameters of these tests with our results because we did not analyze ovulation rate. In the review by Jee, information regarding the efficacy of ablation and sclerotherapy was compared to cystectomy in terms of ovarian reserve, the recurrence rate, and the pregnancy rate [47]. In two randomized studies, similar cumulative pregnancy rate was found while significantly lower rate was observed in the ablation group than in the cystectomy group (23.5% vs. 66.7%, *p* < 0.05 [48]; and 23.3% vs. 59.4%, *p* < 0.05 [49]). Similar results were obtained by Alborzi [50] and Mircea [51]. The authors of this review concluded that more research is needed to demonstrate whether the pregnancy rate is different between ablation and cystectomy.

Our study did not demonstrate an increased pregnancy rate in the volumetric HD group. This could be a consequence of the fact that in the group without HD, a larger number of women planned to conceive. An important element in assessing the impact of endometrioma surgery on the condition of the ovary was the use of Muzzi’s classification. This classification was used only in the work of this author [36]. We did not find differences in histological assessment of ovarian tissues removed with the cysts. But it is worth noting that in the HD group, we did not find ovarian tissue with all types of follicles, i.e., mature ovarian cortex tissue. The assessment of reproductive outcome after HD is scarce in the literature. Our study did not provide convincing evidence, and further research on larger material is required. The strength of our study is the fact of a long‐term 7‐year follow up of the impact of the procedure on reproductive outcome.

## 5. Conclusions

Volumetric HD as an element of endometrioma stripping did not have a significant impact on the data determining the postoperative ovarian reserve and reproductive outcome. Volumetric HD using only neutral fluid for tissue separation allowed for more frequent removal/evacuation of the intact lesion using an endobag. The use of this type of HD reduced the frequency of lesion fragmentation before removal by the trocar. In none of the patients who underwent volumetric HD, removal of mature ovarian cortex tissue along with the cyst was observed.

## Funding

No funding was received for this manuscript.

## Conflicts of Interest

The authors declare no conflicts of interest.

## Supporting Information

We found no statistically significant differences in reproductive outcomes between patients undergoing volumetric hydrodissection and those undergoing classical dissection. However, there was a noticeable, although not statistically significant, higher use of infertility treatments, including ART, in the classical dissection group. We also observed a higher number of pregnancies in the classical dissection group, which may be explained by the fact that these patients were more likely to be planning a pregnancy. On the other hand, the classical dissection group also showed a higher, though not statistically significant, rate of miscarriages.

## Supporting information


**Supporting Information** Additional supporting information can be found online in the Supporting Information section.

## Data Availability

Research data are not shared.
