# Supplementary material for: Reproductive Outcome After Laparoscopic Ovarian Endometrioma Stripping With Volumetric Hydrodissection
Source: Obstet Gynecol Int. 2026 Feb 18;2026:2586905. doi: 10.1155/ogi/2586905 (PMC12914591; doi:10.1155/ogi/2586905)
Supplement: Supplementary file 1 — Supporting Information Additional supporting information can be found online in the Supporting Information section. [file OGI-2026-2586905-s001.docx]

Supplementary

Figure S1. Reproductive outcome seven years after surgery in group of volumetric HD application (N=19) and in group with classical HD use (N=22)

Figure S1. We did not observe differences between the groups in terms of reproductive outcome.
